# Design for Multi-Layer Thermal Protective Clothing Based on Numerical Simulation of Heat Transfer

**DOI:** 10.3390/ma19122478

**Published:** 2026-06-09

**Authors:** Xiaoling Chen, Cunyun Nie

**Affiliations:** 1 School of Future Technology, Hunan Institute of Engineering, Xiangtan 411104, China; 2 School of Computational Science and Electronics, Hunan Institute of Engineering, Xiangtan 411104, China

**Keywords:** thermal protective clothing, textile materials, heat transfer, numerical simulation

## Abstract

It is well-known that high-performance thermal protective clothing is crucial for personnel working in high-temperature environments, such as firefighters. Thermal protective clothing design usually integrates textile materials’ type, thickness, physical and chemical properties (such as thermal conductivity), ergonomics, and environmental adaptability. In this study, the heat transfer process and the optimal thickness are mainly discussed for providing some references on the design of this clothing. The thickness design of thermal protective clothing fabrics is carried out via numerical heat transfer simulations based on experimental data obtained from manikin tests. Firstly, one heat transfer model for thermal protective clothing, including three textile materials’ layers and one air layer, is constructed according to Fourier’s law of heat conduction, Newton’s law of cooling, and the Stefan–Boltzmann law, with appropriate boundary conditions assigned. Secondly, the finite volume element method, which has the important advantage of preserving conservation properties for physical quantities, is employed to discretize the heat transfer model. Thirdly, the convective heat transfer coefficient, which characterizes heat exchange between fluid and solid surfaces, is determined approximately by the least-squares method based on the given data, while the heat transfer process is simultaneously simulated. Fourthly, the thicknesses of the second and fourth layers are critical to the performance of thermal protective clothing. Two optimization algorithms are proposed to determine the optimal thickness configuration that effectively balances thermal insulation and wearing comfort. From the above results, it is recommended to use multilayer textile composite materials incorporating aerogel insulation layers and phase-change material interlayers.

## 1. Introduction

Some people, such as firefighters, are often enforced to carry on some special tasks in high-temperature environments. It is necessary for them to put on some special clothes, such as thermal protective clothing, and to be exempt or lightly harmed from the impact of high temperature [1]. Hence, it is crucial that the safety and reliability of this clothing is guaranteed for them in these high-temperature environments. The textile materials for this clothing should possess some essential functions, such as being waterproof, having thermal insulation, and being breathable. Textile material properties and geometric characteristics are often critical in the design of thermal protective clothing which attributes directly influence the garment’s thermal insulation performance, fit, mobility, and overall protective efficacy [2,3]. One reasonable heat transfer mathematical model and accurate numerical simulation results can guide the design of thermal protective clothing.

There have been many studies on thermal protective clothing over the years. Early on, Torvi established a single-layer heat transfer model for the outer shell material of thermal protective clothing by considering prolonged exposure to different radiation conditions [4]. Subsequently, many scholars have investigated the multi-layer model of heat and moisture transfer in thermal protective clothing. Mell proposed a heat transfer model between layers of multilayer fabrics [5]. Since phase change materials have a certain impact on the effect of thermal protection, Mercer and Elgafy established a multi-layer dynamic heat transfer model containing phase change materials [6,7]. Considering the influence of moisture on the thermal protection effect, Lawson and Ahmed established a heat and moisture transfer model of multilayer fabrics [8,9]. Later, Fu, M. and Wang, Z. presented a complete model of heat and moisture transfer for multilayer firefighter protective clothing, including conductive, two-flux radiative heat transfer, moisture bulk flow, moisture absorption, and desorption and diffusion by fibers [10,11]. Recently, Acharya, J. presented one modified model to assess the thermal protective performance of fire-retardant clothing exposed to both flame and radiant source [12]. Some scholars have also investigated the effects of heat and steam fluxes on skin response, proposed a fractional bioheat model, predicted the burn injury time under prolonged fire exposure, evaluated the performance of the parameters of thermal protective clothing, and discussed heat dissipation [13,14,15]. Cheng established one multiphysics coupling model for a heated textile system including the skin layer, simulating heat conduction, convection, and radiation [16]. As mentioned above, heat transfer models of thermal protective clothing are becoming increasingly mature.

Numerical simulation of thermal protective clothing becomes increasingly significant. It can reduce the testing risks and the costs of research and experimental development and obtain the quantitative analysis on many factors affecting skin burn distribution, such as heat flux and garment thickness, and enables the prediction of burn risks [17]. These numerical simulations typically comprise two types: forward problems and inverse problems, with the temperature distribution or burn risk predictions and the prediction of some parameters of textile materials, respectively.

In recent years, increasing numerical investigations and experimental validations have been carried out. Su et al. separately explored the influence of air gap thickness on thermal protective performance under dry and wet thermal exposure conditions [18]. Pan established a coupled heat transfer numerical model containing phase-change material layers to predict thermal responses under sudden convective and radiant heat flux from pre-flashover fire scenarios [19]. Liang discretized the heat conduction model and analyzed the temporal variation and spatial distribution of skin temperatures under a given working environment [20]. Tian et al. constructed a three-dimensional heat transfer model incorporating the air gap layer inside firefighter’s protective clothing to mitigate skin burn risks in fire environments [21]. Mohammad conducted a comprehensive literature review and analysis of 93 large-scale compartment fire experiments to identify the key factors that affect the HRR required for flashover [22]. Numerical simulations for forward problems usually play a crucial guiding role in material selection and fabric structure design by providing a clear understanding of the heat transfer process.

The inverse design problems of multilayer thermal protective clothing have been well explored with various optimization objectives. Fan et al. proposed an inverse problem of porosity determination with the goal of minimizing heat damage under low-temperature conditions, to meet the requirement of warmth retention [23]. Xu et al. further presented inverse design strategies for textile materials considering comprehensive wearing comfort performance [24]. Cui systematically investigated inverse problems for both single-layer and multilayer fabrics and determined the fabric thickness, porosity, thermal conductivity, and multiple structural and thermal parameters [25]. Recently, Han, R. employed the characteristics of its forward feedback acyclic model through the adjustment of parameters for clothing layer thickness under different environmental temperatures with the use of a neural network [26]. Ruan proposed an algorithm based on PINNs to solve the parameter inversion problem of three-dimensional steady state heat conduction equation [27]. According to numerical investigations and experimental validation for thermal protective clothing, many researchers focused on the design of the textile materials and fabrics. Su characterized thermal protective performance of flame-retardant fabrics exposed to hot steam and low-level thermal radiation [28]. Han, Y. developed one model about the fabric structure of a firefighter’s flame-resistant outer layer, and constructed a geometric and mesh model of fabric air-layer skin [29]. Hence, reliable numerical methods are useful for the design of practical heat protective clothing.

In this paper, we investigate thermal protective clothing composed of three layers with another air layer between the thermal protective clothing and the human skin. One heat transfer mathematical model is established based on the obtained experimental data. The finite volume element method is employed to discretize the proposed heat transfer model. The convective heat transfer coefficient is approximately determined by the least-squares method based on the given data, and the heat transfer process is accurately fitted and simulated. Finally, two optimization algorithms are proposed to determine the optimal thickness configuration that effectively balances thermal insulation and wearing comfort. Numerical results provide valuable references for the practical production of thermal protective clothing and enhance the fundamental understanding of heat transfer phenomena within textile materials.

## 2. Materials

The experimental and numerical data adopted in this study are sourced from the 2018 China Undergraduate Mathematical Contest in Modeling, corresponding to the official topic regarding the optimal structural design of thermal protective clothing for high-temperature working scenarios (The relative data can be downloaded at: https://dxs.moe.gov.cn/zx/a/hd_sxjm_sthb/200601/1610456.shtml accessed on 24 April 2026).

**Figure 1:**
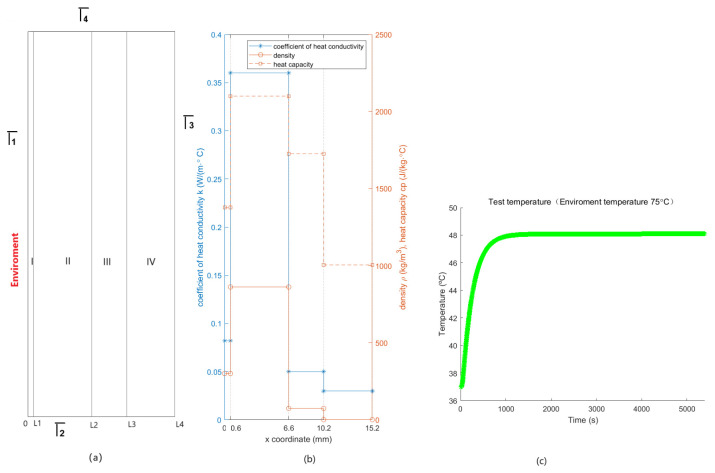
(**a**) Four layers lie in region Ω. (**b**) Some physical parameters for four layers. (**c**) Test temperatures for 5400 moments.

In this study, the thermal protective clothing consists of three functional fabric layers, namely Layer I, Layer II, and Layer III, with another air layer between the thermal protective clothing and the human skin also considered. Their spatial positions along the x-axis, defined as L1, L2, and L3, respectively, are as illustrated in Figure 1a, where $L_{1}=0.06$ mm, $L_{2}=6.6$ mm, and $L_{3}=10.2$ mm are given by the above data. Specifically, Layer I is directly exposed to the high-temperature external environment, while the air gap between Layer III and the human skin is defined as Layer IV whose thickness is 5 mm. Denote $\Omega_{i}$ as the *i*th subregion and the thickness of it as $d_{i}\;,1\leq i\leq 4$, respectively. It is obvious that the computational region $\Omega =\cup_{i=1}^{4}\Omega_{i}$.

The main thermal physical parameters of each layer, including density $\rho_{i}$, specific heat capacity $c_{i}$, and thermal conductivity $\lambda_{i}$, are listed in Table 1 below and illustrated in Figure 1b.

The measured skin temperature data from the manikin test are presented as a scatter plot. It shows the recorded temperature values along the x-axis direction on the outer surface of the dummy skin at 5400 (discrete) moments (as seen in Figure 1c).

The transferability of thermal protective clothing models is significantly affected by environmental and human factors, but effective extrapolation can be achieved through parameter calibration and scenario adaptation. Although the 2018 mathematical modeling competition dataset is based on idealized assumptions, it still can provide a reliable theoretical framework for real-world applications.

## 3. Methods

### 3.1. Mathematical Model

In this subsection, a heat transfer model for thermal protective clothing is established, including model assumptions, governing equations, boundary conditions, and interface thermal contact conditions between adjacent layers.

#### 3.1.1. Model Hypothesis

The following assumptions are adopted in this study:

(1) All fabric layers are assumed to be homogeneous and isotropic with constant thermal conductivity.
(2) Only heat conduction and thermal radiation are considered; moisture transfer and internal heat generation within each layer are neglected.
(3) Heat and mass transfer processes, including water vapor migration and sweat evaporation inside the protective clothing, are neglected in the present model.
(4) The temperature distribution is continuous across adjacent layers, while the temperature gradient exhibits a discontinuous jump at each material interface.
(5) The temperature at the bottom of the fourth air layer is used to characterize the surface temperature of human skin.
(6) The interfacial contact thermal resistance between adjacent layers is ignored, and perfect thermal continuity is assumed at all contact interfaces.
(7) The human skin surface is regarded as an ideal blackbody, with a thermal radiation emissivity equal to 1.0.

#### 3.1.2. Heat Transfer Model

It is impractical to carry on high-temperature exposure tests on human subjects wearing protective clothing, owing to potential safety hazards, high material costs, and large experimental consumption. It is therefore essential to establish an accurate mathematical model to simulate the heat transfer process, which provides theoretical support for the structural and material design of high-performance thermal protective clothing.

According to the model hypothesis in Section 3.1.1, together with the law of conservation of energy and Fourier’s law of heat transfer, one mathematical model is established as follows.

The heat transfer control equation

(1) $$\begin{array}{c} -\rho c\frac{\partial T}{\partial t}-\nabla \cdot \left(\lambda \nabla T\right)=0,\;\;x=\left(x,y\right)\in \Omega , \end{array}$$

where ρ=ρ(x), c=c(x), λ=λ(x), and $\Omega =\overset{4}{\underset{i=1}{⋃}}\Omega_{i}$, $\Omega_{i}$ is the rectangle subregion, and $\partial \Omega =\overset{4}{\underset{i=1}{⋃}}\Gamma_{i}$,  $\Gamma_{i}$ corresponds to left, bottom, right, and up boundary, respectively, shown as Figure 1a, and the subregions from I to IV, respectively, represent four layers: the first, second, and third layers of fabric and the air layer between the third layer and the human skin, and $\rho_{i}$, $c_{i}$, and $\lambda_{i},\;1\leq i\leq 4$ are the heat flux density, heat capacity, and heat transfer coefficient.

The following boundary conditions are embedded within Equation (1)

(2) $$\begin{array}{c} -\lambda_{1}\frac{\partial T}{\partial n}=h_{1}\left(T_{env}-T\left(0,t\right)\right):=g_{1}\left(T,h_{1},t\right),\;\;x\in \Gamma_{1},\\ -\lambda_{3}\frac{\partial T}{\partial n}=h_{2}\left(T\left(L,t\right)-T_{hum}\right):=g_{2}\left(T,h_{2},t\right),\;\;x\in \Gamma_{3},\\ \frac{\partial T}{\partial n}|=0,\;\;x\in \Gamma_{2}⋃\Gamma_{4}, \end{array}$$

where $h_{1},h_{2}$ are, respectively, the convective heat transfer coefficients between the environment and Layer I and between Layer III and human skin, and $T_{env},T_{hum}$ is the temperature of the environment and the human skin, and T(0,t),T(L,t) is the temperature on boundary $\Gamma_{1}$ and $\Gamma_{3}$ at moment *t*, respectively.

The following initial condition is endowed with Equation (1)

$$\begin{array}{c} T\left(x,0\right)=37\;{}^{\circ}C. \end{array}$$

The coordinate system on the computational domain Ω is defined as follows: the origin (0,0) is located at the lower-left corner, while the *x*-axis and *y*-axis lie along the horizontal lower boundary and the vertical left boundary, respectively. The temperature and heat flux density in the norm direction are continuous on the interfaces $x=x_{i}=L_{i},1\leq i\leq 3$ (as seen in Figure 1a),

(3) $$\begin{array}{c} T\left(x_{i}^{-},y,t\right)=T\left(x_{i}^{+},y,t\right),\\ \lambda_{i}\frac{\partial T}{\partial n}\left(x_{i}^{-},y,t\right)=\lambda_{i+1}\frac{\partial T}{\partial n}\left(x_{i}^{+},y,t\right), \end{array}$$

where the signs “−” and “+” in $T\left(x_{i}^{-},y,t\right),T\left(x_{i}^{+},y,t\right)$ (resp. $\lambda_{i}$ and $\lambda_{i+1}$) mean the temperatures (resp. heat transfer coefficient) on the left and right sides of interface $x_{i}=L_{i}$, respectively.

In this paper, we call Equation (1), together with boundary, initial, and interface conditions, as the heat transfer model.

### 3.2. Finite Volume Element Method for the Heat Transfer Model

Numerical discretization of heat transfer governing equations is conventionally implemented via the finite difference method and the finite element method, as widely documented in the existing peer-reviewed literature [11,30]. Differing from the conventional numerical frameworks mentioned above, the present work adopts the finite volume element method to conduct the discrete numerical solution of the established heat transfer theoretical model (1). Notably, the application of the finite volume element method in the numerical simulation of protective fabric heat transfer behavior is rarely reported in relevant research studies, which further highlights the novelty and methodological innovation of the current numerical investigation. On one hand, the finite volume method can preserve the local conservation of physical quantities, making the numerical simulation of heat conduction more physically accurate. On the other hand, another advantage of this method is its flexibility in handling irregular boundary regions. In fact, the regions of human skin and the third layer of thermal protective clothing are often irregular. Hence, this method is more broadly applicable.

**Figure 2:**
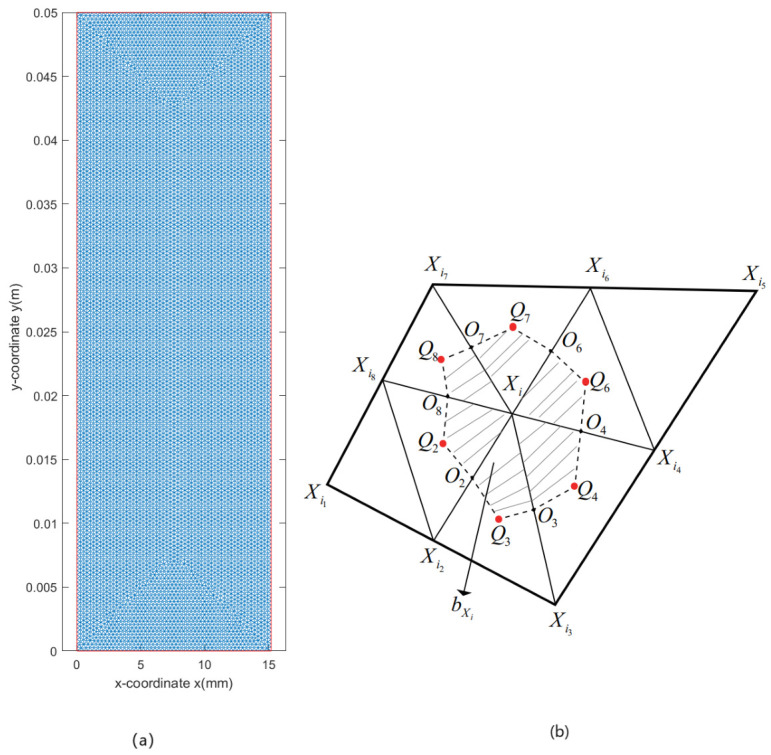
(**a**) The triangular partition $\Omega_{h}$. (**b**) Dual element $b_{x_{i}}$, where Point $X_{i}=x_{i}$, $X_{i_{k}}=x_{i_{k}}$,1≤k≤8.

Let $t_{h}=\left\{t_{i},0\leq i\leq N_{t}\right\}$ be the partition of the interval $\left[t_{0},t_{e}\right]$, and $\delta t_{i}=t_{i}-t_{i-1}=\frac{t_{e}-t_{0}}{N_{t}}$, where $t_{0}$ and $t_{e}$ are the initial and the end moments, respectively. Let $\Omega_{h}=\left\{\tau_{i},1\leq i\leq M\right\}$ denote the triangular partition of the region  Ω, where  *M* is total number of partition elements, and let  $X=\{x_{1},\cdots ,x_{N}\}$ be the set of partition nodes, where  *N* is the total number of partition nodes (as shown in Figure 2a). Let $\Omega_{h}^{*}=\left\{b_{x_{i}},1\leq i\leq N\right\}$ be the dual partition of $\Omega_{h}$, where $b_{x_{i}}$ is the dual element corresponding to Node $x_{i}$ (as seen in Figure 2b), and $O_{i},2\leq i\leq 8,i\neq 5$ is the midpoint of $x_{i}x_{k_{i}}$, and $Q_{k}$ is the corresponding barycenter of the triangle $▵x_{l}x_{i}x_{l-1}$, where $Q_{2}$ is the barycenter of the triangle $▵X_{8}X_{i}X_{2}$.

The backward Euler method is used to discretize the term $\frac{\partial T}{\partial t}$ in Equation (1),

(4) $$\begin{array}{c} \rho cT^{k+1}-\delta t\nabla \cdot \left(\lambda \nabla T^{k+1}\right)=\rho cT^{k},\;\;x=\left(x,y\right)\in \Omega , \end{array}$$

where $T^{k+1},T^{k}$ are, respectively, the temperature of the (k + 1)th and *k*th step, as in $T:=T^{k+1}$.

From (4)

(5) $$\begin{array}{c} \rho cT-\delta t\nabla \cdot \left(\lambda \nabla T\right)=\rho cT^{k},\;\;x=\left(x,y\right)\in \Omega . \end{array}$$

we will employ the linear finite volume element method to discretize Equation (5).

Firstly, we introduce the trial and test function spaces, respectively, as follows:

$$\begin{array}{c} U_{h}=\left\{u_{h}\left(x\right)\in C\left(\bar{\Omega }\right):u_{h}{|}_{\tau_{k}}\in P_{1},\overset{M}{\underset{k=1}{⋃}}\tau_{k}=\Omega_{h}\right\}, \end{array}$$

and

$$\begin{array}{c} V_{h}=\left\{\;v_{h}\left(x\right):v_{h}\left(x\right)\in L^{2}\left(\Omega \right),\;v_{h}{|}_{b_{x_{i}}}=constant,b_{x_{i}}\in \Omega_{h}^{*}\right\}, \end{array}$$

where $v_{h}\left(x\right)=\left\{\begin{array}{cc} 1, & \;\;\forall \;x\in b_{x_{i}},\;\\ 0, & \;\;\forall \;x\notin b_{x_{i}}. \end{array}\right.$

From the Gauss Theorem and the conditions (3), the discretization variational form of Equation (5) is written as follows: To find $T_{h}\in V_{h}$ such that

(6) $$a\left(T_{h},v_{h}\right)=f\left(v_{h},T_{h}^{n}\right),\;\;\forall T_{h}\in U_{h},\;\;\;T_{h}^{0}=T\left(x,0\right),$$

where

$$\begin{array}{cc} a(T_{h},v_{h}) & =\sum_{i=1}^{N}\left(\int_{b_{x_{i}}}\rho cT_{h}v_{h}dx-\int_{\partial b_{x_{i}}}\lambda \frac{\partial T_{h}}{\partial n}v_{h}ds\right)-\int_{\Gamma_{1}}g_{1}\left(T_{h},h_{1},t\right)v_{h}ds-\int_{\Gamma_{3}}g_{2}\left(T_{h},h_{2},t\right)v_{h}ds,\;\;\\ f(v_{h},T_{h}^{n}) & =\sum_{i=1}^{N}\int_{b_{x_{i}}}\rho cT_{h}^{n}v_{h}dx. \end{array}$$

In the above formulation, the convective heat transfer coefficients $h_{1},h_{2}$ are explicitly defined by Equation (2). By substituting these prescribed coefficients into the variational framework, both the continuous heat transfer model and its discrete finite volume element approximation are thereby rigorously established and well posed.

In certain practical scenarios where the specific values of $h_{1},h_{2}$ are not readily available or require empirical determination, corresponding numerical algorithms can be strategically designed to identify or estimate these key parameters. This supplementary estimation procedure ensures the generality and applicability of the proposed numerical scheme in real-world engineering situations where experimental data are incomplete.

### 3.3. The Least-Squares Method for Convective Heat Transfer Coefficients

In this subsection, the discrete heat transfer model (6), together with the temperature data acquired on the outer basal layer of the skin at discrete time $t_{i}$, is adopted to estimate the two unknown parameters $h_{1},h_{2}$. For simplicity and computational feasibility, $h_{1},h_{2}$ are assumed to be constant.

The least-squares mathematical model is written as follows:

(7) $$\left({\hat{h}}_{1},{\hat{h}}_{2}\right)=arg\min_{h_{1},h_{2}}\sum_{i=1}^{N}{\left[T_{h}\left(L,t_{i};h_{1},h_{2}\right)-T^{*}\left(t_{i}\right)\right]}^{2},$$

where ${\hat{h}}_{1},{\hat{h}}_{2}$ are the estimators, and $T^{*}\left(t_{i}\right)$ is the measured temperature on the outer base layer of the skin along the direction which is perpendicular to the skin surface, at moment $t_{i}$, and the approximate temperature $T_{h}\left(L,t_{i};h_{1},h_{2}\right)$ in (7) satisfies the heat transfer model (6).

To obtain the optimal values of $h_{1},h_{2}$, we present the least-squares algorithm as follows, referred to as Algorithm 1.
**Algorithm 1** Obtaining $h_{1},h_{2}$Step 1: Input initial iterative values $h_{1},h_{2}$. Step 2: Obtain the temperatures $T_{h}\left(L,t_{i};h_{1},h_{2}\right)$ in the region Ω by solving the model problem (6) from $t_{0}$ to $t_{i}$. Step 3: Obtain the new ${\tilde{h}}_{1},{\tilde{h}}_{2}$ by solving the minimum problem (7). Step 4: Update the values of $h_{1},h_{2}$ by ${\tilde{h}}_{1},{\tilde{h}}_{2}$. Step 5: Compute the residual $res={\left(\sum_{i=1}^{N}{\left[T_{h}\left(L,t_{i};h_{1},h_{2}\right)-T^{*}\left(t_{i}\right)\right]}^{2}\right)}^{\frac{1}{2}}$,               if res>tol then                       goto Step 2,               endif              where $tol=10^{-8}$ is the tolerance of the error. Step 6: Output the optimal $h_{1},h_{2}$. Subsequently, solve the discrete heat transfer model (6) again to obtain the corresponding optimal temperature distribution.

With Algorithm 1, the following results are obtained.

**Figure 3:**
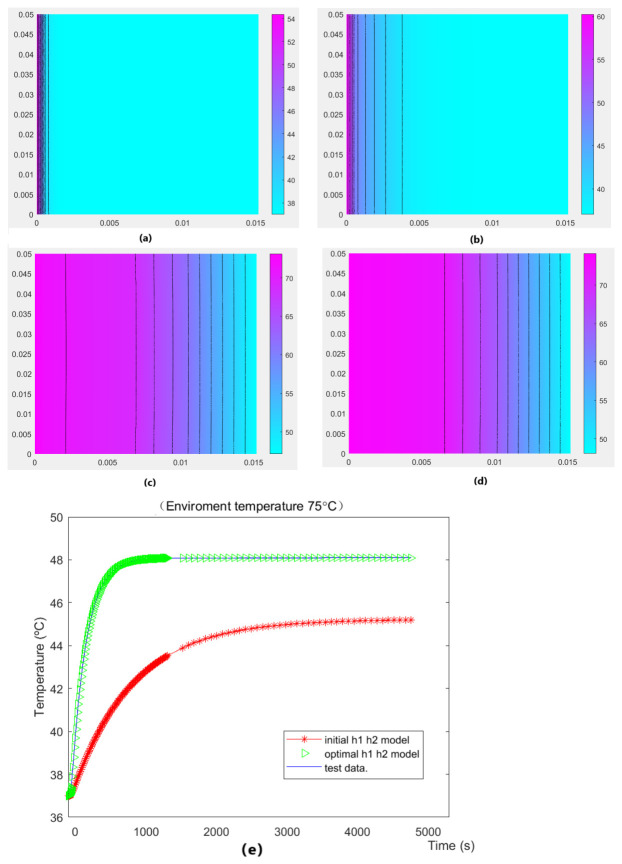
(**a**–**d**): Temperature distributions at 4 typical moments. (**e**) The comparison temperature distribution of different time outside the skin along the x-axis direction on the initial $h_{1}=h_{2}=10$ model, the optimal $h_{1}=119.2745,h_{2}=8.9775$ model, and the given test data.

(1) The optimal parameter $h_{1}=119.2745,h_{2}=8.9775$, which corresponds to the optimal heat transfer model corresponding to the given data.
(2) The temperature distributions at four typical moments in the computational region Ω are shown in Figure 3a–d when the environmental temperature is 75 °C. From the figures, one can find that the temperature distribution is symmetric, which is solved by the finite volume element method (6).
(3) Although the investigated heat transfer problem can be simplified into a one-dimensional form in ideal conditions, both the continuous governing model (1) and the discrete numerical model (6) are universally applicable to arbitrarily shaped computational domains, including complex curved boundaries that conform to real human body surface geometries. This highlights a prominent advantage of the finite volume element method in effectively handling irregular geometric boundaries in practical thermal engineering scenarios.
(4) Some comparison of temperature distributions fitting results along the x-axis direction are shown in Figure 3e. From it, one can find that the initial parameter $h_{1}=h_{2}=10$ does not agree with the given test data, while the optimal one $h_{1}=119.2745,h_{2}=8.9775$ is remarkably agreeable with the least-squares method, and the minimum residual is 0.577.

Accordingly, the proposed Algorithm 1 is verified to be numerically reliable and computationally efficient. This algorithm provides a feasible and effective technical approach for the accurate inversion of unknown convective heat transfer coefficients in thermal protective clothing heat transfer simulations.

**Remark** **1.** *Convective heat transfer coefficients $h_{1},h_{2}$ exhibit moderate to high sensitivity to noise in experimental temperature data, with the degree of influence depending on the heat transfer stage and the airtightness of the clothing system [31].*

In summary, the complete continuous theoretical model and discrete numerical heat transfer model have been fully constructed and validated in the present work. On the basis of the verified thermal model, the subsequent optimization design of protective fabric material thickness can be further carried out in the following sections.

### 3.4. Optimization Design on the Thickness of Fabric Materials

In this paper, we consider three layers of heat protective clothing and the air layer. It is known that the first (outer) layer of it is usually composed of some metal aluminum foil composite material, which can reflect thermal radiation more than 70%. The second layer, called the insulation layer, is composed of aramid fiber or glass fiber material whose thermal stability is excellent. The third layer, called the comfort layer, possesses some functions, such as breathability, flame-retardant fabric, and temperature-rising control. The air layer is charged with the radiative heat transfer with the skin surface, which directly affects the perceived temperature.

Based on the established heat transfer model, this subsection mainly focuses on the thickness optimization of the second and fourth fabric layers. These two layers play a decisive role in the overall protective performance, and thus are the key objectives in the structural design of thermal protective clothing.

#### 3.4.1. Mathematical Model for the Optimal Thickness $d_{2}$

It is assumed that all physical parameters, initial conditions, boundary conditions, and interface conditions are predetermined. Meanwhile, the thicknesses of Layer I, Layer III, and Layer IV are fixed, while only the thickness of Layer II is taken as the design variable. On this premise, we aim at determining the minimum optimal thickness of the fabric material for Layer II under a series of reasonable constraint conditions, which are summarized as follows:

(1) The steady-state temperature on the outer base side of the skin: *T_hum_* ≤ 47 °C.
(2) The high temperature operation time $t_{e}$ = 1 h (unit h: hour).
(3) The time exceeding 44 °C should be less than 5 min (unit min: minute).
(4) $0.6\;\mathrm{mm}\leq d_{2}\leq 25\;\mathrm{mm}$ (unit mm: millimeter).
(5) *T_env_* = 65 °C.

According to the requirements above, one single objective optimization model can be constructed as follows.

Optimization objectives: $\min d_{2}$.

Optimization requirements:

(8) $$\left\{\begin{array}{c} \max_{0\leq t\leq 60}T\left(L,t;d_{2}\right)\leq 47\;{}^{\circ}C,\\ \min\{t:T\left(L,t;d_{2}\right)\leq 44\;{}^{\circ}C\}\geq 55\;\min,\\ 0.6\;\mathrm{mm}\leq d_{2}\leq 25\;\mathrm{mm},\\ T_{env}=65\;{}^{\circ}C, \end{array}\right.$$

where $T(L,t;d_{2})$ satisfies with the heat transfer model (6).

**Figure 4:**
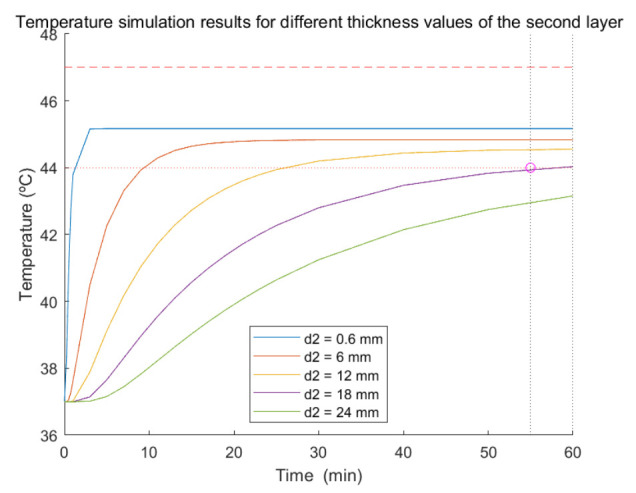
Temperature distributions at different moments for the skin surface with different $d_{2}$.

**Figure 5:**
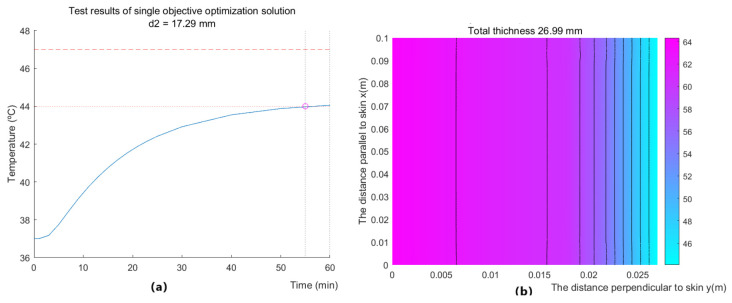
(**a**) The optimal $d_{2}$ and temperature distribution in 60 min. (**b**) The optimal $d_{2}$ and temperature distribution at the end time in Ω.

Firstly, one can find that if other parameters are fixed, the temperature on the outer side of the skin is a monotonically nondecreasing function with respect to heat transfer time, and at steady state, the skin temperature decreases monotonically with respect to $d_{2}$. Hence, we can transform it to solve critical value problems that satisfy constraint conditions. The sequential quadratic programming (SQP) algorithm is applied for this nonlinear optimization problem, together with the finite volume element method (6) for the heat transfer model. Numerical results are obtained and shown in Figure 4 and Figure 5.

(1) The approximate minimum $d_{2}$ is shown in Figure 4, where one can find that the minimum thickness of $d_{2}$ is about 18 mm from the four test choices $d_{2}=0.6,6,12,18,24$ (unit is mm), respectively. It satisfies all the above requirements. Of course, one also can find that all the temperatures are below 44 °C as $d_{2}=24\;\mathrm{mm}$, and it is too thick to increase the weight of the heat protective clothing and not convenient enough to work and also increases the cost, although it is the best one in the sense of numerical value. Hence, the best choice of thickness $d_{2}$ is near 18 mm after careful considerations.
(2) The optimal thickness $d_{2}=17.29\;\mathrm{mm}$ is obtained by the SQP algorithm. For this optimal $d_{2}$, we also carry on the simulation for the heat transfer model (1). The corresponding temperature distributions are shown in Figure 5a,b. As seen in Figure 5a, the requirement $\min\{t|T\left(L,t;d_{2}\right)\leq 44\;{}^{\circ}C\}\geq 55\;\min$ is successfully satisfied. Furthermore, Figure 5b illustrates that the temperature of heat protective clothing approaches the environmental temperature 65 °C, while the skin-perceived temperature is controlled for the optimal requirements. In this case, the total thickness of this heat protective clothing is 26.99 mm.

#### 3.4.2. The Optimal Combination Between $d_{2}$ and $d_{4}$

In the following section, we will focus on designing the thickness of the protective clothing by comprehensively considering multiple objectives.

(1) To be comfortable, the thickness of the clothing should be as minimal as possible so that it is lightweight and convenient to operate in while achieving the same insulation effect.
(2) To reduce costs, the thickness of the second layer should be as minimal as possible, because the fourth layer is the air layer with no cost.
(3) To guarantee excellent performance stability of the thermal protective clothing, a reasonable balance between $d_{2}$ and $d_{4}$ should be achieved. On one hand, an excessively thick air layer may cause non-uniform thermal distribution, local overheating, and even skin burn risks. In addition, the thermophysical properties of the air interlayer are easily affected by complex environmental factors, such as human sweat and water vapor. On the other hand, the second insulation layer cannot be designed too thin, since it serves as the core functional layer to block external thermal radiation and maintain effective thermal insulation.
(4) The optimized thickness scheme should fully satisfy practical engineering requirements, including the allowable maximum skin temperature and the effective high-temperature working duration.

Based on the objectives mentioned above, we construct the following multi-objective optimization model.

Optimization objectives:

$$\left\{\begin{array}{c} \min d_{2}+d_{4},\\ \min d_{2}. \end{array}\right.$$

Optimization requirements:

(9) $$\left\{\begin{array}{c} \max_{0\leq t\leq 30}T\left(L,t;d_{2}\right)\leq 47\;{}^{\circ}C,\\ \min\{t|T\left(L,t;d_{2}\right)\leq 44\;{}^{\circ}C\}\geq 25\;\min,\\ 6\;\mathrm{mm}\leq d_{2}\leq 25\;\mathrm{mm},\\ 0.6\;\mathrm{mm}\leq d_{4}\leq 6.4\;\mathrm{mm},\\ T_{env}=80\;{}^{\circ}C, \end{array}\right.$$

where $T(L,t;d_{2})$ satisfies the heat transfer model (6).

**Figure 6:**
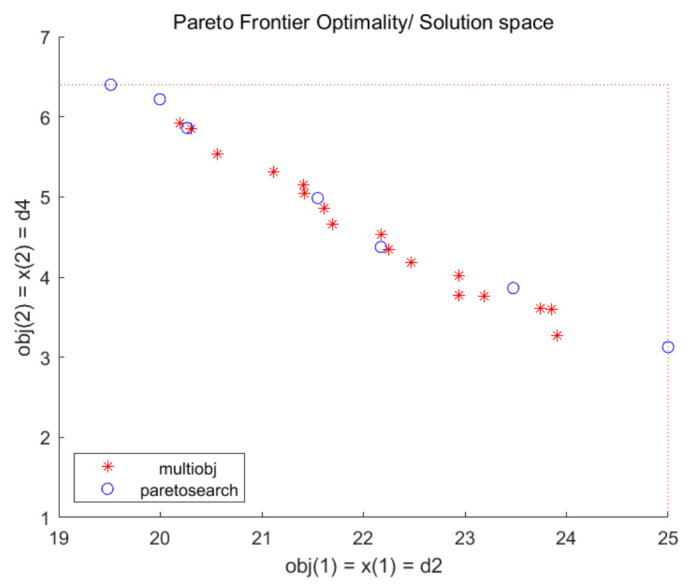
Comparison with two methods for some feasible solutions for $d_{2}$ and $d_{4}$.

**Figure 7:**
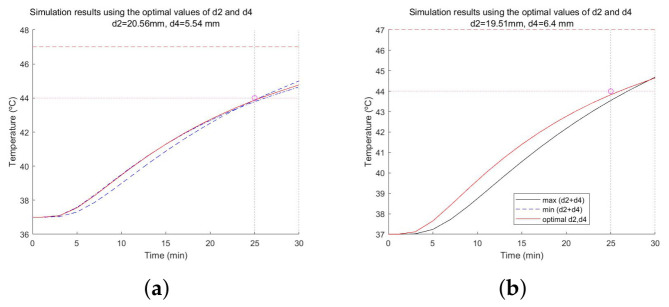
Three cases of temperature distribution from $t_{0}$ to $t_{e}$ (the maximum $d_{2}+d_{4}$, minimum sum $d_{2}+d_{4}$, and optimal $d_{2}$ and $d_{4}$): (**a**) MOGA algorithm. (**b**) PSA algorithm.

**Figure 8:**
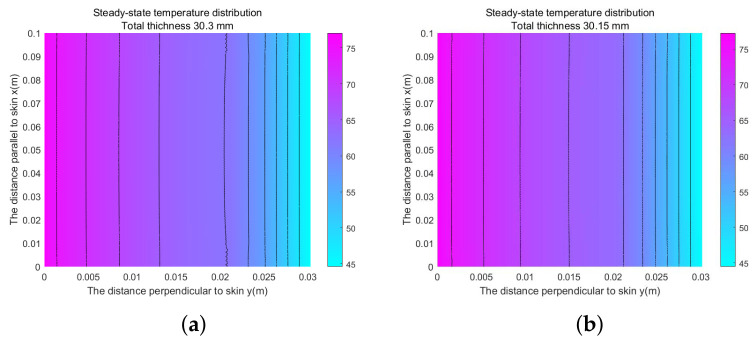
Temperature distribution at $t_{e}$ for optimal $d_{2}$ and $d_{4}$): (**a**) MOGA algorithm. (**b**) PSA algorithm.

We employ two typical algorithms for this optimization problem: Multi-objective genetic algorithm (MOGA), Pareto search algorithm (PSA), respectively, and the finite volume element method (6) for the heat transfer model. The corresponding multi-objective optimization results are shown as Figure 6, Figure 7 and Figure 8.

(1) As displayed in Figure 6, one can find that there are two groups of feasible solutions for $d_{2}$ and $d_{4}$ in the Pareto Frontier optimal solution space, solved by MOGA and PSA, respectively, which confirms the solvability of the optimization problem.
(2) In Figure 7a, obtained by MOGA, one can find three curves of the temperature distribution from $t_{0}$ to $t_{e}$: maximum $d_{2}+d_{4}$ (blue line), minimum sum $d_{2}+d_{4}$ (red line), and optimal $d_{2}$ and $d_{4}$ (red line). The optimal choice: $d_{2}=20.56\;\mathrm{mm}$, $d_{4}=5.54\;\mathrm{mm}$. These three lines agree with all the requirements of the optimization problem, and for the yellow line, the temperature increases the fastest after 25 min because it is thinnest. This phenomenon is consistent with the actual problem, which verifies the correctness of the computation and simulations. Figure 7b is similar, and the optimal choice is as follows: $d_{2}=19.55\;\mathrm{mm}$, $d_{4}=6.4\;\mathrm{mm}$. Comparing with Figure 7a,b, the temperature distribution of two optimal choices are nearly uniform, but the cost of case (b) is more agreeable because of the thinner $d_{2}$ layer.
(3) For the optimal $d_{2}$ and $d_{4}$, we carry on the simulation for this heat transfer model. The corresponding temperature distribution is shown in Figure 8a,b. From the figures, the requirement $\min\{t|T\left(L,t;d_{2}\right)\leq 44\;{}^{\circ}C\}\geq 25\;\min$ is reached when the environmental temperature is $T_{env}=80\;{}^{\circ}C$, and the highest temperature is less than 47 °C. Hence, we can provide some optimal methods for adjusting the optimal thickness $d_{2}$ and $d_{4}$ to satisfy the different temperature requirements.

**Remark** **2.** *Although the thickness above is inappropriate from the perspective of ergonomics, it provides a mathematical model and method for thickness design based on experimental data.*

## 4. Results

The main numerical and optimal results obtained in this work are summarized systematically as follows.

(1) Using the least-squares method, we have obtained the optimal heat convective transfer coefficients $h_{1}=119.2745,h_{2}=8.9775$ to best fit the given data.
(2) The optimal thickness $d_{2}=17.29\;\mathrm{mm}$ is obtained by the SQP algorithm. The corresponding temperature distributions show that the requirement $\min\{t|T\left(L,t;d_{2}\right)\leq 44\;{}^{\circ}C\}\geq 55\;\min$ is reached. One can find the temperature of heat protective clothing is near the environmental temperature 65 °C, while the skin-perceived temperature is controlled for the optimal requirements. The total thickness of this heat protective clothing is 26.99 mm.
(3) The optimal choices: (1) $d_{2}=20.56\;\mathrm{mm}$, $d_{4}=5.54\;\mathrm{mm}$. (2) $d_{2}=19.55\;\;\mathrm{mm}$ and $d_{4}=6.4\;\;\mathrm{mm}$ are solved by MOGA and PSA, respectively. They agree with all the requirements of the optimization problem, and temperature distributions are consistent with the actual problem, which verifies the correctness of the computation and simulations.
(4) A complete multi-layer coupled heat transfer theoretical model including three-layer thermal protective clothing and one air layer is established with reasonable physical interface conditions and realistic thermal boundary conditions. Subsequently, the finite volume element method is introduced and applied to discretize the proposed continuous heat transfer equations, ensuring numerical stability and computational efficiency.

Under the assumptions in Section 3.1.1, the overall simulation and optimization results finally provide a series of feasible optimized thickness configurations for layered fabric structures, which can effectively achieve a comprehensive balance between high-efficiency thermal insulation performance, lightweight wearing comfort, and low economic manufacturing cost in practical thermal protective clothing design.

## 5. Discussion

The obtained results above are reasonable under the conditions stated in this study. However, since some of the assumptions presented in this study are not suitable in real-world scenarios, several problems are worth further discussing and investigating, such as some conditions in the assumptions, textile materials and their geometric, physical, and chemical Characteristics, and temperature thresholds for thermal protective clothing.

### 5.1. Some Assumptions in the Heat Transfer Model

The assumptions (2) and (3) in Section 3.1.1 can be revised in the following study. Excluding the effects of moisture on heat conduction and steam burn risks will severely underestimate the deviation in thermal protective performance under real-world conditions, leading to distorted optimization results in firefighter turnout gear design or firefighting strategies. In actual fire environments, moisture is inevitable [32]. Factors such as firefighter perspiration, wet turnout gear, and the generated steam during firefighting can significantly alter heat transfer dynamics:

(i) More complex heat conduction situations. Moisture increases the fabric’s thermal conductivity and heat capacity, meaning that under the same radiant heat exposure, a wet turnout suit transfers heat to the skin layer more rapidly. Ignoring this factor results in a significantly higher thermal protection performance (e.g., TPP value) in simulations or tests than what is actually available, creating a dangerous safety misjudgment.
(ii) Steam burn risk. In high-temperature conditions, moisture vaporizes, and steam can penetrate clothing layers and then condense on the skin and release latent heat, causing “secondary burns.” This injury mechanism is independent of direct flame contact. If steam risk is not included in optimization models, a critical injury pathway is overlooked, leaving a fatal flaw in protective design.

Another assumption that the model assumes perfect thermal contact between layers may also not be reasonable. Introducing interfacial thermal resistance significantly alters the predicted temperature distribution, causing the inner layer temperature to rise faster, thereby reducing the actual effective protective performance of thermal protective clothing and making the optimization results more representative of real-world conditions.

### 5.2. The Textile Materials and Their Characteristics

The heat transfer mathematical models in thermal protective clothing must integrate the multi-layer synergistic effects of woven fabrics, non-woven insulation layers, and waterproof membranes to achieve a balance between efficient thermal insulation and high thermophysiological comfort. From the structural and functional perspective of textile materials, each layer plays a distinct role in the model.

(i) Woven fabric outer layer. Serving as the first line of defense, this layer is typically made from flame-resistant fibers such as aramid or PBI, offering high strength, abrasion resistance, and protection against radiant heat. In the mathematical model, its low thermal conductivity (approximately 0.04–0.06 W/m·K) and high reflectivity are input as boundary conditions to slow down the initial rate of heat ingress from external flames or radiant sources.
(ii) Non-woven insulation layer. This layer acts as the core thermal barrier of the protective system. As described in patent CN-223100179-U, its porous structure effectively traps air, reducing both convective and conductive heat transfer. In modeling, it is often treated as a porous medium, with an effective thermal conductivity model (e.g., Maxwell–Eucken equation) introduced to describe the relationship between porosity and thermal resistance. A high porosity (>80%) can reduce the thermal conductivity to below 0.03 W/m·K, significantly enhancing insulation performance.
(iii) Waterproof and breathable membrane layer. Materials such as TPU or ePTFE membranes provide both water resistance and water vapor permeability. In coupled heat–moisture models, this layer must simultaneously account for moisture diffusion flux and thermal conduction resistance. Its micro-porous structure allows water vapor to pass through (improving comfort) while blocking liquid water intrusion. Research indicates that composite fabrics incorporating this membrane maintain better steam protective performance under wet conditions compared to traditional porous materials in international academic contexts.

Additionally, to enhance thermophysiological comfort, modern thermal protective clothing often integrates phase change materials (PCM) or moisture-regulating fibers in the inner layer. These components delay the skin temperature rise by absorbing latent heat during phase transitions or modulating microclimate humidity. Their functions require the introduction of a transient heat capacity term and humidity-dependent heat–moisture transfer equations into the model for dynamic simulation.

In designing thermal protective clothing, it is not only essential to understand the aforementioned functions of textile materials, but also their geometric, physical, and chemical properties. In the field of thermal protection, considering only layer thickness is insufficient; the structural characteristics, areal mass, porosity, and chemical composition of textile materials must be comprehensively evaluated, as these factors collectively determine the actual thermal insulation performance and wear comfort of protective clothing [33].

(i) Structural characteristics. The fabric’s weaving pattern (e.g., plain, twill, and satin) or web-forming process (e.g., needle-punching and hydro-entangling) directly influences thermal resistance distribution and mechanical strength. For instance, a 3D hollow structure can create more stagnant air layers, significantly enhancing thermal insulation while reducing weight.
(ii) Areal mass. Areal mass is positively correlated with heat capacity: the greater the areal mass, the slower the temperature rise when absorbing the same amount of heat, resulting in stronger thermal buffering. However, excessive areal mass increases garment load, impairing mobility, so a balance between protection and lightweight design is essential.
(iii) Porosity. Non-woven materials with high porosity (>80%) effectively trap air, reducing convective and conductive heat transfer. Their porous structure can be regarded as a low-conductivity medium, with thermal conductivity potentially dropping below 0.03 W/m·K, making it key to achieving efficient insulation.
(iv) Material composition. The chemical nature of fibers determines their inherent flame resistance and thermal stability. Aromatic polymers such as aramid and polyimide, with rigid ring structures, have limiting oxygen index (LOI) values as high as 29–38%, exhibiting self-extinguishing properties. In contrast, flammable fibers like polyester and nylon require flame-retardant modification to enhance safety.

Moreover, the synergistic effects among layers in a composite structure cannot be overlooked: the outer layer reflects radiant heat, the middle layer blocks conductive heat, and the inner layer regulates the microclimate. Relying solely on thickness-based design while ignoring the coupled influence of these parameters will lead to an overestimated TPP value (Thermal Protective Performance) and reduced actual protection capability.

Finally, the ergonomic requirements for thermal protective clothing are also crucial, including static fit, dynamic range of motion, operational convenience and personal protective equipment (PPE) compatibility, localized pressure and discomfort mitigation, thermal–moisture comfort, safety and biocompatibility, and lightweight design.

### 5.3. Some Temperature Thresholds for Thermal Protective Clothing

In thermal conduction models for thermal protective clothing, the temperature thresholds of 44 °C and 47 °C correspond to the critical skin tolerance limit and the onset of second-degree burns, respectively, with their determination based on human skin thermal injury physiology and international protection standards (e.g., NFPA 1971) [34].

The temperature threshold 44 °C is the upper limit for prolonged skin contact tolerance. Exceeding this temperature triggers sustained heat stress, leading to cellular metabolic disruption. In TPP testing, this point is commonly used as the criterion for “acceptable heat load,” guiding the optimization of thermal comfort in protective clothing for extended firefighting operations.

The temperature threshold 47 °C marks the onset temperature for second-degree burns, where exposure exceeding one second can cause damage to both the epidermis and dermis. This threshold is used to evaluate safety margins under extreme short-term thermal exposure and serves as the core indicator for the “minimum compliance requirement” of protective performance. The choice of threshold significantly impacts optimal thickness design:

When 44 °C is the limiting criterion, the model favors a balance between insulation and lightweight design, yielding optimized thicknesses typically in the range of 3.0–4.0 mm. This is suitable for routine firefighting tasks, emphasizing the wearer’s endurance and operational sustainability. When 47 °C is the limiting criterion, the model prioritizes maximizing thermal resistance, recommending increased thicknesses of 4.5–5.5 mm. This sacrifices some flexibility to achieve high-level protection against instantaneous flame or radiant heat, commonly applied in high-risk scenarios such as industrial arc flashes or explosion rescue missions. Thus, the selection of temperature thresholds reflects a fundamental safety strategy: lower thresholds ensure safety during prolonged operations, while higher thresholds address acute, high-intensity thermal threats. In practice, multi-objective optimization must be employed to align garment design with specific mission requirements.

### 5.4. Future Research

In the follow-up research, on the basis of the existing deterministic heat transfer model, environmental random interference factors such as humidity, sweat erosion, and repeated thermal aging of fabrics will be further introduced to construct a stochastic thermal protection optimization model, so as to further improve the environmental adaptability and service durability of the optimized protective clothing scheme. We will add three factors as follows.

(i) Introduce a coupled heat–moisture transfer model for fabrics. In clothing layer modeling, incorporate the fiber’s moisture adsorption/desorption process, and consider its dynamic impact on thermal conductivity and heat capacity. For instance, when fabric moisture content increases, thermal conductivity can rise by two or three times. This variation must be embedded as a variable into the heat conduction equation, rather than assuming constant thermal resistance.
(ii) Improve the human thermal regulation model to include sweat evaporation and accumulation mechanisms. Adopt an enhanced Stolwijk based sweat model to calculate the actual skin surface evaporation efficiency constrained by ambient humidity and air velocity. When relative humidity exceeds 80%, evaporation efficiency significantly decreases, leading to a rise in perceived temperature.
(iii) Construct a risk submodule for steam penetration and condensation heat release in high-temperature and high-humidity environments, water vapor can penetrate clothing and condense within inner layers, releasing latent heat (approximately 2259 kJ/kg), causing “secondary burns.” A steam diffusion and phase-change heat release term should be added to the model, dynamically solved in conjunction with microclimate layer humidity changes, which is especially critical in scenarios involving sealed protective garments.

We will next clarify the structure and type of textile materials used in the protective clothing, and integrate heat conduction models that account for moisture and steam, to carry on related experiments and research under realistic and acceptable conditions.

## 6. Conclusions

Based on the above results and discussion, some conclusions can be drawn as follows.

(i) The identification of convective heat transfer coefficients is a typical inverse problem. This paper proposes a least-squares fitting method for these coefficients, and machine learning algorithms can also be considered in subsequent research.
(ii) In the proposed heat transfer model with the given conditions in this paper, the optimal thickness of $d_{2}$ and $d_{4}$ for heat protective clothing is obtained by some optimization algorithms, and the corresponding results are reasonable in this case, which can provide some references on the relative researches. However, there is still a certain gap between these results and practical application scenarios. For example, some factors such as the influence of water vapor on the heat transfer, the ergonomic requirements of protective clothing, and the structure and properties of fabric materials, should be incorporated into the heat transfer model in the following study, which will make numerical results more consistent with actual working conditions.
(iii) For numerical simulations of the heat transfer model, the finite volume (element) method has been widely favored by many researchers, because it can flexibly handle complex boundaries and maintain local conservation of physical quantities. In contrast, neural network methods, such as back propagation (BP) neural networks, physics-informed neural networks (PINNs) and so on, have the advantages of no requirement for mesh generation and embedding governing equations into the residual losses, which deserves our more attentions in future research.

## Figures and Tables

**Table 1:** The thermal physical parameters of each layer: ρ, *c*, and λ.

Parameters	$\Omega_{1}$	$\Omega_{2}$	$\Omega_{3}$	$\Omega_{4}$
$\rho_{i}$	300	86.2	74.2	74.2
$c_{i}$	1377	2100	1726	1005
$\lambda_{i}$	0.082	0.037	0.035	0.028

## Data Availability

The original contributions presented in this study are included in the article. Further inquiries can be directed to the corresponding authors.

## Abbreviations

The following abbreviations are used in this manuscript:

PCM	phase change material
SQP	sequential quadratic programming
MOGA	multi-objective genetic algorithm
PSA	Pareto search algorithm
